# *In Vitro* Investigation of the Antimicrobial Properties of Gerês Propolis in Bacteria Isolated from Companion Animals and Safety Profile Characterization Using the *Galleria mellonella* Model

**DOI:** 10.3390/pathogens14080826

**Published:** 2025-08-21

**Authors:** Rafael Rodrigues, Rui Almeida, Soraia C. V. Rodrigues, Joana Castro, Ricardo Oliveira, Nuno Mendes, Carina Almeida, Sónia Silva, Daniela Araújo, Cristina Almeida-Aguiar

**Affiliations:** 1 Department of Biology, University of Minho, 4710-057 Braga, Portugal; pg55652@alunos.uminho.pt (R.R.); pg55715@alunos.uminho.pt (R.A.); soraiarodrigues2001@gmail.com (S.C.V.R.); nuno.rafael@hotmail.com (N.M.); 2 INIAV—National Institute for Agrarian and Veterinary Research, Rua dos Lagidos, Lugar da Madalena, 4485-655 Vairão, Portugal; joana.castro@iniav.pt (J.C.); ricardo.oliveira@iniav.pt (R.O.); carina.almeida@iniav.pt (C.A.); soniasilva@ceb.uminho.pt (S.S.); 3 Centre of Biological Engineering, University of Minho, 4710-057 Braga, Portugal; 4 LEPABE—Laboratory for Process Engineering, Environment, Biotechnology and Energy, Faculty of Engineering, University of Porto, 4200-465 Porto, Portugal; 5 ALiCE—Associate Laboratory in Chemical Engineering, Faculty of Engineering, University of Porto, 4200-465 Porto, Portugal; 6 LABBELS—Associate Laboratory, University of Minho, 4710-057 Braga, Portugal; 7 CBMA—Centre of Molecular and Environmental Biology, Department of Biology, University of Minho, 4710-057 Braga, Portugal

**Keywords:** Gerês propolis, antimicrobial activity, companion animals, *Galleria mellonella*, toxicity

## Abstract

Propolis, also known as bee glue, is a natural resinous mixture produced by Western honeybees and has long been recognized for its potential therapeutic properties. Recent research has focused on its diverse bioactivities, particularly its antimicrobial effects against a broad spectrum of microorganisms, including human but also animal pathogens. However, further investigation is required to fully elucidate the pharmacological properties and potential toxicity of propolis to support its broader application. This study investigated the antimicrobial efficacy and safety of an ethanol extract of Portuguese propolis collected from the Gerês region (G23.EE). The antimicrobial activity was assessed *in vitro* against both Gram-positive and Gram-negative bacteria associated with infections in companion animals, using the agar dilution method. To evaluate potential toxicity, the extract was administered via injection and topical application in an in vivo *Galleria mellonella* larval model, with health parameters monitored over a 96 hours period. The *in vitro* results showed that G23.EE was more effective against Gram-positive bacteria, including *Staphylococcus* spp. (e.g., *S. felis*, *S. hominis*, *S. simulans*, and *S. pseudintermedius*; MIC = 0.5 mg/mL) and *Enterococcus faecium* (MIC = 1.5 mg/mL), than against Gram-negative bacteria, such as *Escherichia coli* and *Klebsiella oxytoca* (MIC > 8.0 mg/mL). No significant adverse effects were observed in *G. mellonella* larvae following injection or brushing with propolis extract concentrations up to 8.0 mg/mL. Overall, these findings suggest that Portuguese propolis from Gêres is a promising, safe, and effective natural antimicrobial agent for targeting Gram-positive bacterial infections in companion animals.

## 1. Introduction

Bacterial infections represent a substantial challenge affecting both human and animal health, with a particularly high prevalence among companion animals [1]. Certain pathogens, such as *Escherichia coli* and *Staphylococcus* spp., are capable of zoonotic transmission [2], posing risks to both animal and human health through direct contact. Furthermore, infections caused by antibiotic-resistant bacteria (ARB) are a growing concern in clinical veterinary settings [3].

Recent studies conducted between 2021 and 2023 identified *Staphylococcus* spp. (Gram-positive) and *Klebsiella* spp. (Gram-negative) as predominant etiological agents of infections in domestic cats and dogs [4,5]. *Staphylococcus pseudintermedius* was the most frequently isolated *Staphylococcus* spp. from pets, mostly from cases of otitis and skin infections, with 36% of isolates exhibiting broad antimicrobial resistance [4]. *Klebsiella* spp., particularly *Klebsiella pneumoniae* and *Klebsiella oxytoca*, were also frequently isolated from skin wounds, respiratory tract infections, and urinary infections in pets [5]. Some isolates have been identified as multidrug-resistant (MDR) strains [5], and Ribeiro et al. [6] reported ~20% isolates in a case-series study of *Klebsiella*-induced infections in 697 livestock and companion species animals.

Antibiotics have historically been vital for both treatment and prophylaxis in clinical and agricultural settings, including the animal-derived food industries [7]. However, the widespread and often inappropriate use of antibiotics has contributed to the global emergence of ARB and, critically, MDR strains [8]. These infections now contribute to more than 20,000 deaths annually, representing a growing public health threat, as highlighted by the World Health Organization (WHO) [8,9,10]. These data reinforce the need for proper use of drugs in therapy approaches in domestic animals.

Another major consequence of excessive antibiotic use is the excretion of significant quantities of these compounds—often in active or resistant forms—through feces and urine. Current wastewater treatment methods are frequently inadequate to fully eliminate antibiotic residues [11], allowing these contaminants to persist in the environment [12]. This persistence poses severe health risks, as residues may re-enter the food chain through animal or human consumption [13], disrupting microbiomes and promoting ARB/MDR development in both hosts [14]. Furthermore, resistant strains can spread across species and ecosystems [15,16], adding to the estimated USD 2 billion economic burden of ARB/MDR infections annually in the human healthcare sector alone [17]. These risks highlight the importance of the One Health framework, which emphasizes the interconnectedness of human, animal, and environmental health.

Given these complex challenges and the global rise in ARB/MDR infections, there is an urgent need for accessible, biocompatible, targeted antimicrobial therapies. However, most alternative treatments are often limited, particularly in low- and middle-income countries [18]. Consequently, there is a growing interest in alternative, preferably natural, sources of antimicrobial agents, especially in veterinary medicine, given the significant role animals play in zoonotic transmission [19].

Propolis, a natural resinous product synthesized by honeybees from plant-derived substances such as buds and exudates, combined with salivary enzymes and beeswax, serves as a protective material for hives [20]. The chemical composition of this beehive product varies significantly depending both on the geographical origin of the hive and the local flora, which contributes to the existence of multiple types [21]. Despite variations in chemical composition and, consequently, biological properties, propolis generally exhibits a broad spectrum of bioactivities [21], such as antibacterial [22], antifungal [23], antiviral [24], antiparasitic [25], anti-inflammatory [26], antiproliferative [27], and antioxidant [28] properties.

Propolis sourced from Gerês, a region in Northern Portugal, has demonstrated notable consistency in its chemical fingerprinting and antimicrobial activity over time [29], making this organically sourced propolis particularly attractive for diverse applications [29,30]. However, the efficacy of any antimicrobial agent is closely tied to its biocompatibility and safety profile [31,32]. Consequently, toxicological evaluation is essential prior to a therapeutic application to identify potential adverse effects [33,34].

Given the critical need for novel, natural, and biocompatible antimicrobial agents to combat infections in animals, and thereby mitigate the dissemination of ARB/MDR pathogens across species and environments, propolis from Gerês stands out as a promising candidate. In this study, we investigated the therapeutic potential of this Gerês propolis for veterinary applications by assessing its antimicrobial activity *in vitro* against clinically isolated bacteria from companion animals. Additionally, its in vivo toxicity was evaluated using the *Galleria mellonella* model, a cost-effective and ethically favorable alternative for preliminary in vivo toxicity screening. This model was employed to establish safe and effective dosage levels.

## 2. Materials and Methods

### 2.1. Propolis Sample and Extract Preparation

Propolis from Gerês (G) (41°45′41, 62″ N; 7°58′03, 34″ W), in Northern Portugal, was collected in 2023 (G23) and used in this study. According to the procedure described by Caetano et al. [30], a 15 g G23 sample was extracted via maceration with absolute ethanol (ITW Reagents, S.R.L., >99.9%, Italy, Milano) in a ratio of 1:8 (*w*/*v*) for 24 h at 25 °C and 125 rpm (Orbital Shaker SO1, Bibby, Stuart, UK). Briefly, after a first extraction and subsequent filtration, the residues were re-extracted with the same solvent and filtered under equal conditions. The filtrates were assembled and dried using a rotary evaporator (Buchi R-200, Marshall Scientific, Hampton, NH, USA) at 38–40 °C, 40 rpm, and 120 mbar. The extract obtained—G23.EE—was kept at 4 °C, protected from light, for later use. The yield of extraction was calculated using the formula $\frac{W_{dry\;extract}}{W_{raw\;propolis}}\;\times \;100$, where *W* = weight.

Solutions of the desired concentrations were prepared with absolute ethanol for the antimicrobial assays. For toxicity assays with *G. mellonella*, G23.EE was freeze-dried (Vertical Normal Lab Freeze Dryer YR05190, Kalstein, Paris, France) at −70 °C for 2 days, resulting in a lyophilized (_L_) extract (G23.EE_L_), and solutions of the desired concentrations were prepared with PBS (0.08% NaCl, 0.02% KCl, 0.02% K_2_HPO_4_, and 0.0285% NaHPO_4_) from stock solutions (100 mg/mL and 160 mg/mL) in DMSO (Sigma-Aldrich, >99.7%, Darmstadt, Germany).

### 2.2. Chemical Characterization of Propolis Extract G23.EE

#### 2.2.1. Total Polyphenol Content (TPC)

The colorimetric Folin–Ciocâlteu method [35] was employed to assess the total polyphenol content (TPC), as previously described [30]. Briefly, 40 μL of Na_2_CO_3_ (Merck, Darmstadt, Germany) 7.5% (*w*/*v*), 50 μL of Folin–Ciocâlteu reagent (Sigma-Aldrich, Darmstadt, Germany) (1:10 dilution), and 10 μL of G23.EE were added per well to a 96-well microplate to obtain final G23.EE concentrations ranging between 50 and 350 μg/mL. A blank was prepared for each concentration using 90 μL of solvent and 10 μL of G23.EE, as well as a control with 10 μL ethanol, 40 μL of Na_2_CO_3_, and 50 μL of Folin–Ciocâlteu reagent. After 1 h of incubation in the dark at room temperature (RT, 25 °C), absorbance values were read at 760 nm (Infinite M Plex, Tecan, Männedorf, Switzerland). Total phenolic content was determined using a calibration curve constructed with gallic acid (98%, Thermo Scientific, Porto Salvo, Portugal) as the standard (concentration range: 1–30 μg/mL, R^2^ = 0.97). Results were obtained by linear regression interpolation, and TPC values were expressed as mg GAE/g G23.EE (milligrams of gallic acid equivalents per gram of propolis extract).

#### 2.2.2. Total Flavonoid Content (TFC)

Total flavonoid content (TFC) was quantified in G23.EE with the aluminum chloride (AlCl_3_) colorimetric method [36], as described before [30]. In this assay, 50 μL of 2% AlCl_3_ solution (Acros Organics, Waltham, MA, USA) and 50 μL of G23.EE were added to each well of a 96-well microplate, resulting in a G23.EE concentration range of 200–1600 μg/mL. Blank samples were made with 50 μL of G23.EE at each tested concentration and 50 μL of solvent. The control contained 50 μL of ethanol and 50 μL of AlCl_3_ solution. After incubation at RT for 1 h, absorbance was read at 420 nm. TFC values were determined by linear regression interpolation of a quercetin (Sigma Aldrich, >95%, Darmstadt, Germany) calibration curve, prepared with concentrations in the range of 5-200 μg/mL (R^2^ = 0.99), and they were expressed as mg QE/g G23.EE (milligrams of quercetin equivalents per gram of extract).

### 2.3. Microbial Strains and Growth Conditions

G23.EE antimicrobial properties were determined against a panel of commonly used antimicrobial susceptibility-indicator strains (Gram-positive bacteria *Bacillus cereus* ATCC 7064, *Bacilllus subtilis* 48886, *Staphylococcus aureus* ATCC 6538, Methicillin-Resistant *Staphylococcus aureus* M746665 (MRSA), *Staphylococcus epidermidis* CECT 4/83/ATCC 35983, *Bacillus megaterium*, and the Gram-negative bacterium *Escherichia coli* CECT 423) for comparison purposes with the antimicrobial spectra of other ethanol extracts of propolis from Gerês (G.EEs) [29,30].

Upon validation of G23.EE antimicrobial spectrum, a set of 26 bacterial strains isolated from companion animals and belonging to the collection of microorganisms of the National Institute for Agrarian and Veterinary Research (INIAV) (Table 1) was used to assess the antimicrobial potential of Gerês propolis.

Bacterial strains were subcultured in Luria-Bertani (LB) broth (Grisp Research Solutions, Porto, Portugal) and incubated at 37 °C, with shaking at 200 rpm until reaching an optical density (OD) at 600 nm of 0.4–0.6.

#### Antimicrobial Assays

The antimicrobial potential of G23.EE was evaluated by determining its minimum inhibitory concentration (MIC) values against the above-referenced microbial strains, which belong to the microbial panel commonly used to characterize other G.EEs spectra [29,30], as well as against bacterial isolates from companion animals (Table 1) through the agar dilution method [29]. For that, antimicrobial assays were performed in LBA medium (LB medium supplemented with agar (VWR, BDH Chemicals, Radnor, PA, USA 2% (*w*/*v*)) for bacteria in the absence and in the presence of increasing concentrations of G23.EE: 0.05, 0.1, 0.2, 0.5, 0.75, 1.0, 1.5, and 2.0 mg/mL. Bacterial isolates from Table 1 were also tested in the presence of 4.0 and 8.0 mg/mL G23.EE. LBA containing absolute ethanol, matched to the maximum volume of the extract, was used as control. Three 5 µL drops of each microbial culture (with an OD_600nm_ of 0.4–0.6, as described in Section 2.3) were spotted onto the assay media. Plates were incubated at 37 °C for 24 h. Following incubation, microbial growth was assessed visually. The MIC of G23.EE was defined as the lowest concentration at which no visible bacterial growth was observed. All assays were performed in triplicate across at least three independent experiments.

### 2.4. Galleria Mellonella Larvae

*G. mellonella* larvae were kept at 25 °C in the dark and fed on a pollen-based diet before use. Last instar larvae weighing approximately 250 mg were used for the experiments.

#### Toxicity Assays

Gerês propolis toxicity was assessed by direct injection of G23.EE_L_ into the hemolymph of *G. mellonella* larvae, through the last left proleg previously sanitized with 70% (*v*/*v*) ethanol and using a micro syringe (BD Micro-fine^TM,^ Quirumed S.L.U., Valencia, Spain, and via topical application on the back of each larva. For each assay, 10 larvae were either injected or brushed with 10 μL of propolis at different concentrations (0.01, 0.05, 2, 6, and 8 mg/mL), obtained through dilution of G23.EE_L_ stock solutions (100 mg/mL and 160 mg/mL in DMSO) in PBS. For the control group, 10 larvae were injected/brushed with PBS only. After injection/topical application, larvae were placed back in Petri dishes, in the dark, at 37 °C. Specifically, in the G23.EE_L_-treated larvae, each solution was reapplied every 24 h for the following 3 days. Survival was monitored for 4 days, and the *G. mellonella* health index was assessed every 24 h based on activity (larvae movement), cocoon formation, melanization, and survival, according to the score system created by Loh et al. [37]. Larvae were considered dead if they showed no movement after stimulation. All experiments were performed in triplicate, and at least three independent assays were performed.

### 2.5. Statistical Analysis

Propolis chemical profiling via colorimetric assays and antimicrobial tests were conducted in triplicate and repeated independently a minimum of three times. Results were expressed as mean ± standard deviation (SD). Data from the antimicrobial and toxicity assays are expressed as the mean ± SD of at least three independent experiments, each with three replicates. The results of toxicity assays were compared using ANOVA analysis with Holm–Sidak’s multiple comparisons tests and a confidence level of 95%. For the *G. mellonella* model, Kaplan–Meier survival curves were plotted, and differences in survival were calculated by using the log-rank Mantel–Cox statistical test. All tests were performed using GraphPad Prism 6^®^ (GraphPad Software, Solana Beach, CA, USA).

## 3. Results

### 3.1. Extraction, Chemical Characterization, and In Vitro Antimicrobial Activity of Propolis Extract G23.EE

Propolis G23 was extracted with absolute ethanol to obtain the ethanolic extract G23.EE, with a yield of 70.6%, which was used in antimicrobial assays following confirmation of its broad-spectrum activity (Table S1—Supplementary Materials). G23.EE was further freeze-dried to obtain the lyophilized extract G23.EE_L_ (yield = 73.2%) and used for in vivo toxicity studies.

The chemical composition of propolis has a significant influence on its biological activities, which are most frequently attributed to phenolic compounds, particularly flavonoids [38]. Thus, the chemical characterization and quantification of total phenolic and total flavonoid compounds in propolis extracts using colorimetric analysis methods are common practices [38,39]. The TPC of G23.EE was found to be 142.57 ± 5.60 mg GAE/g G23.EE, while the determined TFC was 28.34 ± 1.98 mg QE/g G23.EE.

The antimicrobial potential of G23.EE was first evaluated against a panel of bacterial strains commonly used to characterize other G.EEs [30] (Table S1—Supplementary Materials), revealing a similar antimicrobial spectrum, namely a higher activity against Gram-positive bacteria [29,30,40].

Further testing of G23.EE against clinical isolates from companion animals showed heightened susceptibility among several Gram-positive strains (Table 2).

The most susceptible isolates to G23.EE were *Staphylococcus felis* J35-B, *Staphylococcus hominis* A11, *Staphylococcus pseudintermedius* A17-2 and A45, and *Staphylococcus simulans* V9-A (MIC = 0.5 mg/mL). G23.EE exhibited a MIC value of 1 mg/mL against other strains of this genus, namely *Staphylococcus condimenti* V16-A and *S. aureus* J63, whereas a higher MIC (1.5 mg/mL) was observed against *Enterococcus faecium* 414 and 434, *Staphylococcus arlettae* CRIO-2, *S. pseudintermedius* J6-B and J43-B, and *Staphylococcus sciuri* J14-B.

Thus, the majority of tested Gram-positive bacteria were susceptible to G23.EE, apart from the isolates *Enterococcus faecalis* 406 and *S. canis* 786, which appear to be more resistant to propolis. All the Gram-negative strains tested (*Escherichia coli* and *Klebsiella oxytoca*), as well as *E. faecalis* 406 and *Streptococcus canis* 786, can be considered resistant to G23.EE within the concentrations tested (up to 8 mg/mL, the maximum).

### 3.2. In Vivo Evaluation of Propolis Toxicity Using the Galleria Mellonella Model

**Figure 1:**
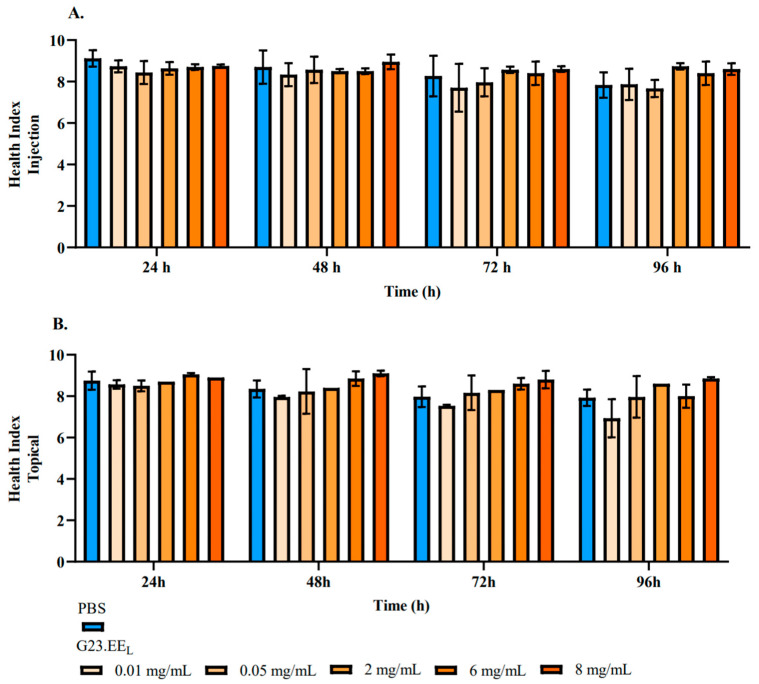
Effects of Gerês propolis administration on the health index of *Galleria mellonella* larvae. Health index of larvae (**A**) injected and (**B**) brushed with 0.01, 0.05, 2, 6, and 8 mg/mL of G23.EE_L_**.** The data were collected over 96 h after infection. As a control, one set of larvae was injected/brushed with PBS only.

The toxicity of G23.EE was evaluated using the *G. mellonella* larvae model, a well-established alternative to mammalian systems for preclinical toxicology [41,42,43,44,45]. *G. mellonella* larvae were injected and brushed with G23.EE_L_, and the larvae health index results remained statistically similar across the G23.EE_L_-treated and G23.EE_L_ non-treated organisms (*p* > 0.05) for both administration routes (Figure 1). Furthermore, there were no significant differences in the health of the larvae between those injected (Figure 1A) and those brushed (Figure 1B) with G23.EE_L_ in all the tested concentrations (*p* > 0.05).

**Figure 2:**
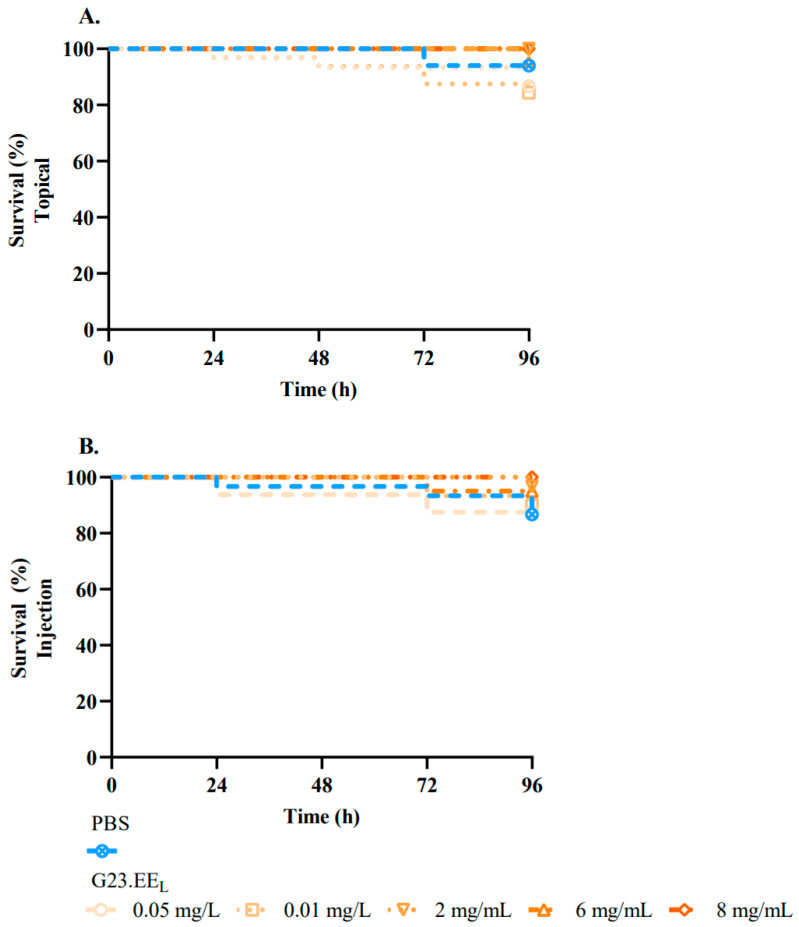
Effects of Gerês propolis administration on the survival of *Galleria mellonella* larvae. Survival of *G. mellonella* after injection (**A**) and brushing (**B**) with G23.EE_L_ at 0.01, 0.05, 2, 6, and 8 mg/mL for 96 h. PBS was used as a negative control.

Survival larvae curves were generated and are presented in Figure 2. No significant larval mortality was registered following injections (Figure 2A) or brush applications at all concentrations of the propolis tested (Figure 2B) when compared to the negative control (PBS).

## 4. Discussion

The extraction of propolis, as with many other plant-derived natural products, is a common procedure, as this beehive product cannot be used in its raw form. Ethanolic extraction is a widely accepted method for isolating bioactive compounds from propolis, as it effectively dissolves phenolic constituents, key components responsible for its antimicrobial and antioxidant activities [46,47]. Previous studies have demonstrated that extraction efficiency increases with ethanol concentration [48,49,50], supporting the use of absolute ethanol in this study. Alternative extraction methods, such as *Soxhlet* extraction at elevated temperatures (e.g., 60 °C), can enhance the yield of flavonoid and phenolic compounds [49,51] highly connected with antimicrobial and antioxidant propolis bioactivities; however, such approaches may compromise compound stability due to thermal degradation and co-extraction of undesirable waxes [51,52]. To preserve the chemical integrity and bioactivity of G23.EE, a milder extraction method was employed, involving maceration and evaporation at temperatures not exceeding 40 °C [50,53].

Following extraction, G23.EE was freeze-dried, resulting in a lyophilized extract (G23.EE_L_) with a yield of 73.2%. Lyophilization has been shown to enhance the release of bioactive components [54] and improve extraction yield [55,56]. The resulting G23.EE_L_ was subsequently used for chemical characterization, as well as *in vitro* antimicrobial testing and in vivo toxicity studies.

The antimicrobial potential of G23.EE against bacterial strains commonly used to characterize Gerês-derived ethanolic extracts (G.EEs) [30] revealed a similar antimicrobial spectrum (Table S1) [29,40,57,58]. Notably, the MIC values of G23.EE against *B. cereus*, *B. subtilis*, MRSA, and *E. coli* match those reported over a decade ago [30]. The greater susceptibility of Gram-positive bacteria is a well-documented feature, observed not only for G.EEs but also for propolis worldwide [21]. Importantly, all G.EEs tested to date have consistently shown strong activity against *B. subtilis*, with MIC values ranging from 0.05 to 0.1 mg/mL. This pattern has remained stable for almost 15 years. This long-term consistency is considered a distinguishing feature of Gerês propolis, suggesting a notable degree of biochemical consistency [29]. While some variability in susceptibility can occur with individual strains, such differences generally fall within the previously reported range [29,30,40]. For example, *S. aureus* ATCC 6538 showed a slightly reduced sensitivity to G23.EE (MIC = 2 mg/mL) compared to G11.EE and G15.EE [30]. These variations in strain susceptibility may reflect environmental or geographical influences, such as wildfires or beehive relocations [57].

Among the various bioactivities attributed to propolis, antimicrobial activity is particularly noteworthy. Comparative studies have established a strong correlation between the total polyphenol and flavonoid contents and the antimicrobial activity of propolis extracts [59,60]. TPC and TFC were determined for G23.EE in order to provide a general chemical characterization of this extract, and showed to be, respectively, 142.57 ± 5.60 mg GAE/g G23.EE and 28.34 ± 1.98 mg QE/g G23.EE. The TPC value obtained for G23.EE is in the range of the values registered for other ethanol extracts of propolis from Gerês and from earlier years (107.96 ± 5.6 to 226.73 ± 4.3 mg GAE/g extract; 2011–2022) [29,30] and similar to the values obtained for other Portuguese propolis samples [30]. The TFC of G23.EE is slightly lower than the contents previously reported for Gerês propolis (from 31.0 ± 1.3 to 51.7 ± 0.9 mg QE/g of extract), yet in the same range. However, it is marginally higher than the values observed for other European poplar-type propolis samples [61]. Despite the variations that can be mainly attributed to changes in the flora visited by the bees throughout the years, as well as to uncontrollable natural events, such as wildfires, this general consistency of TFC and TPC values over the years indicates the stability of bioactive compounds over time. Delving further into the chemical composition of this natural product, acacetin, apigenin, caffeic acid, caffeic acid isoprenyl ester (CAIE), 3,4-dimethyl-caffeic acid (DMCA), chrysin, galangin, kaempferide, kaempferol, *p*-coumaric acid, *p*-coumaric acid methyl ester, pinobanksin, and ferulic acid have been reported as the main phenolic compounds in propolis from Gerês [29]. Notably, these compounds have been consistently detected in samples collected over multiple consecutive years, indicating a strong and stable chemical profile [29]. Taking into account this consistency in G.EEs chemical profiles together with their relatively uniform antimicrobial spectra, and the pattern of TFC and TFC values, it is reasonable to infer that G23.EE likely shares a similar phenolic composition. In fact, the antimicrobial spectrum of G23.EE (Table S1), as well as its total phenolic content and total flavonoid content, are in agreement with the established chemical characteristics of Gerês propolis and show stability for more than a decade.

Focusing on the results of the antimicrobial properties of the G23.EE against bacteria isolated from companion animals, the most susceptible bacteria were *S. felis* J35-B, *S. hominis* A11, *S. pseudintermedius* A17-2 and A45, and *S. simulans* V9-A (MIC = 0.5 mg/mL; Table 2). These results align with prior reports of European propolis efficacy against Gram-positive bacteria, specifically *Staphylococcus* isolates, with MIC values commonly ranging between 0.032 and 0.512 mg/mL [62]. Notably, MRSA isolates (J63) also demonstrated susceptibility to G23.EE (Table 2), showing its relevance as an alternative antimicrobial agent [63,64]. The genus *Staphylococcus* comprises Gram-positive bacteria and is among the most prevalent pathogenic agents affecting humans, being recognized for its multidrug resistance [4]. Currently, several *Staphylococcus* spp. strains have been identified as responsible for infections in the auditory and urinary tracts, as well as in the open wounds of companion animals [65]. These strains are capable of being transmitted to humans through contact with infected animals, subsequently surviving in the new host and developing resistance to both veterinary and human-use antibiotics, becoming MDR [1,4]. In this context, the antimicrobial activity of G23.EE against these isolated strains becomes highly relevant.

A MIC of 1 mg/mL of G23.EE was found for other species of the genus *Staphylococcus*, namely *S. condimenti* V16-A and *S. aureus* J63 (Table 2). *S. condimenti* can be found in the gastrointestinal tract of some species and is occasionally isolated from fermented foods; it is considered part of the normal microbiota in companion animals [66]. Unlike certain clinical *Staphylococcus* isolates and some food-derived strains, *S. condimenti* typically lacks notable resistance traits, but its involvement in human infections highlights its potential opportunistic pathogenicity [67]. In contrast, *S. aureus* is a major human and animal pathogen with a well-documented history of multidrug resistance [68]. It is the most virulent of the common staphylococcal species, frequently responsible for skin infections and capable of causing more severe conditions, such as pneumonia, endocarditis, and osteomyelitis [69]. Thus, the moderate susceptibility observed in these two strains to G23.EE/propolis further supports the therapeutic potential of this natural product. Indeed, MRSA was susceptible to a green propolis ethanolic extract (MIC of 0.2463 mg/mL) [63], and another MRSA strain (*S. aureus* ATCC 43300) showed susceptibility to a brown propolis ethanolic extract from Romania (MIC = 0.1 mg/mL) [64].

Strains for which G23.EE displayed MIC values of 1.5 mg/mL included *E. faecium* 414 and 434, *S. arlettae* CRIO-2, *S. pseudintermedius* J6-B and J43-B, and *S. sciuri* J14-B (Table 2). *Enterococcus faecium* is a naturally occurring commensal bacterium within the gastrointestinal tract of humans and other mammalian species, and it is widely distributed in the environment. It has been implicated in urinary tract infections, meningitis, and bacteraemia [70], while *S. arlettae* isolated from the skin of mammals and birds revealed resistance to novobiocin [71]. *S. pseudintermedius* is an opportunistic pathogen that secretes immune-modulating virulence factors [72], has many adhesion factors, and has the potential to form biofilms. These characteristics are important determinants of its recognized zoonotic potential and bacterial pathogenicity [73]. *Staphylococcus schleiferi* V3-B has been identified as a causative agent of otitis externa, otitis media, and pyoderma in both dogs and cats [74,75,76]. This strain has already been documented in human infections, involving conditions such as surgical site infections, pediatric meningitis, or endocarditis [77]. Its resistance to β-lactam antibiotics has been reported, contributing to persistent and recurrent infections in both human and veterinary contexts [78]. Lastly, the *S. sciuri* species group, now classified as *Mammaliicoccus sciuri* [79], comprises five species primarily regarded as commensal bacteria associated with diverse habitats, including humans, animals, and the environment [80]. These species, while often commensal, are increasingly implicated in opportunistic infections in companion animals. Notably, members of this group have been shown to harbor multiple virulence and antibiotic resistance genes, including those involved in biofilm formation and toxins linked to toxic shock syndrome, resembling gene profiles found in *S. aureus* [81].

All the Gram-negative strains tested (*E. coli* and *K. oxytoca*) were found to be resistant to G23.EE within the concentrations tested (up to 8 mg/mL, the maximum; Table 2). This resistance is likely due to the protective outer membrane typical of Gram-negative bacteria, which acts as a barrier to the penetration of many natural compounds [82,83]. The lower susceptibility of *E. coli* to propolis is well known; for example, one study determined a MIC value of 5 mg/mL for Irish and Czech ethanolic extracts of propolis only when they were synergistically combined with antibiotics, such as ceftriaxone [61].

Most tested Gram-positive bacteria were susceptible to G23.EE, apart from the isolates *E. faecalis* 406 and *S. canis* 786 (Table 2). *E. faecalis* is a widely distributed species in the environment and commonly inhabits the gastrointestinal tract of humans and other mammals as a commensal organism [84]. It can act as an opportunistic pathogen, causing urinary tract infections, meningitis, and bacteraemia, particularly in health care conditions [85,86]. The limited susceptibility of *E. faecalis* 463 to G23.EE (MIC = 8 mg/mL) and resistance of *E. faecalis* 406 (MIC > 8 mg/mL) are consistent with the high resistance of these species to β-lactam antibiotics and their involvement in nosocomial infections [87]. The detection of *E. faecalis* and other *Enterococcus* spp. in animals, including companion and livestock animals, has raised increasing concern regarding their role in veterinary medicine, particularly with respect to antimicrobial resistance [88]. In the same way, *S. canis* is a bacterial species first isolated in dogs and is recognized as a multi-host pathogen capable of causing diseases of varying severity, including bacterial infections, in a wide range of mammals, including humans [89]. Its zoonotic potential underscores its relevance in both veterinary and human medicine, and the increasing reports of antimicrobial resistance in *S. canis* further highlight the need for surveillance and a One Health approach to manage infections associated with this species [90,91].

Considering the susceptibility of the tested Gram-positive isolates to G23.EE and taking into consideration that most of these isolates were recovered from ear and skin infections in companion animals, the local application of propolis could be considered as a therapeutic treatment. This possibility is further supported by the G23.EE activity against bacterial strains previously identified as MDR (Table 2), including *S. aureus* J63, *S. hominis* A11, *S. pseudintermedius* A17-2, and J43-B [4], highlighting the potential of this natural extract as a new, natural, simpler, and less costly alternative to conventional antibiotics, thereby helping to prevent the emergence of new MDR species [92].

The agar dilution method was selected over a liquid-based assay for quantitively assessing microbial susceptibility due to several methodological advantages, which are particularly important when working with plant-derived extracts. The use of a solid medium facilitates direct visual observation of microbial growth, which is essential for accurately determining the minimum inhibitory concentration (MIC). Additionally, this approach overcomes challenges associated with testing complex mixtures containing both soluble and poorly soluble components, as well as oily or strongly colored plant-based natural products [93,94]—an important consideration in the present study. Moreover, the agar dilution method allows for the simultaneous testing of multiple bacteria on the same plates [95].

The use of invertebrate models for in vivo toxicity testing has become more widespread due to the ethical, economic, and logistical complications of some vertebrate models [96]. Widely accepted in both academic and industrial settings, the larvae of the *G. mellonella* model offer several advantages, such as a short life cycle, ease of maintenance, cost-effectiveness, and suitability for testing at mammalian body temperatures, and they are particularly useful for screening novel therapeutic agents before progressing to mammalian testing [41,44,45]. They have been used for pathogenicity studies of microorganisms [43,97], as well as in toxicity tests for drugs and natural products [41,42,98].

*G. mellonella* larvae health index of G23.EE_L_-treated and non-treated organisms showed no statistically significant differences regardless of propolis administration routes (Figure 1). Yet, no significant differences were observed between the health of the larvae injected (Figure 1A) and those brushed (Figure 1B) with G23.EE_L_ (*p* > 0.05). This means that, at the concentrations tested and under both application methods, propolis did not reveal any significant level of toxicity, which is similar to what was reported by Pedrinha and colleagues [99]. These findings are consistent with those of other studies, including one involving an Iranian ethanolic propolis extract, which was found to be non-toxic by in vivo rodent assays when administered at a dose of 2000 mg/kg [100]. Similarly, propolis extracted in Croatia showed an attenuation of alloxan-induced hepatotoxicity and nephrotoxicity in mice, thus further supporting the safety of this natural product [101].

Survival larvae curves showed no significant larval mortality following G23.EE_L_ injection (Figure 2A) or brush application at all the tested concentrations (Figure 2B). Besides the previous report concerning the absence of significant propolis toxicity in the rodent in vivo model [100], a more recent study using the *G. mellonella* model also described no significant toxicity of Brazilian ethanol-extracted propolis injected into larvae [99], but at a much lower concentration (3 mg/mL). In another study, not only was the absence of toxicity of propolis demonstrated in ethanol extract from Southern Brazil, but it was also able to retain its bioactivity after gastric digestion [102]. Altogether, our findings and these recent studies highlight the safety and biocompatibility of this natural compound.

## 5. Conclusions

This study confirms the antimicrobial properties and the safety profile of the ethanolic extract of propolis collected from Gerês in 2023. The extract G23.EE demonstrated substantial activity against Gram-positive bacteria isolated from companion animals, including several multidrug-resistant (MDR) strains. These findings position G23.EE as a viable candidate for the development of alternative or adjunctive treatments for superficial infections, especially those affecting the skin and ears of companion animals, even when antimicrobial-resistant bacteria are involved. The absence of toxic effects in the *G. mellonella* model further supports the biocompatibility and clinical potential of G23.EE, namely for veterinary applications.

Importantly, by combining antimicrobial testing against animal-associated bacteria with in vivo toxicity assessment in *Galleria mellonella* model, our study presents a comprehensive evaluation of both the efficacy and safety of Gerês propolis in a way not previously reported. These contributions help to expand the biological relevance and potential therapeutic application of Gerês propolis, particularly in veterinary contexts, and support future translational research.

While G23.EE showed limited activity against Gram-negative bacteria, this outcome is consistent with known limitations of propolis and highlights the need for further investigation. Future work should explore higher extract concentrations (>8 mg/mL), synergistic combinations with other propolis types, or adjunct use with conventional antimicrobials.

In summary, G23.EE emerges as a promising, safe, clean-sourced, biocompatible, and natural antimicrobial agent for veterinary use, with potential broader applications. Continued exploration of such natural products is essential to help address the global challenge of antimicrobial resistance.

## Figures and Tables

**Table 1:** Bacterial species, isolates, and sources and origin from companion animals tested for G23.EE susceptibility.

Bacterial Species	Isolate	Animal	Origin of the Isolate
Gram-positive			
*Enterococcus faecalis*	406	Dog	Spleen (corpse)
	463	Dog	Liver (corpse)
*Enterococcus faecium*	414	Dog	Spleen (corpse)
	434	Cat	Blood culture (corpse)
*Streptococcus canis*	786	Dog	Blood culture (corpse)
*Staphylococcus arlettae*	CRIO-2	Cat	Ear infection
*Staphylococcus aureus*	J63	Cat	Skin infection
*Staphylococcus condimenti*	V16-A	Dog	Ear infection
*Staphylococcus felis*	J35-B	Cat	Skin infection
*Staphylococcus hominis*	A11	Cat	Skin infection
*Staphylococcus pseudintermedius*	A17-2	Dog	Skin infection
	A45	Dog	Ear infection
	J6-B	Dog	Ear infection
	J43-B	Dog	Skin infection
*Staphylococcus schleiferi*	V3-B	Dog	Skin infection
*Staphylococcus sciuri*	J14-B	Dog	Skin infection
*Staphylococcus simulans*	V9-A	Dog	Skin infection
Gram-negative			
*Escherichia coli*	A36	Cat	Gastrointestinal
	A28	Cat	Skin infection
	D22-A	Cat	Skin infection
	J6-A	Dog	Ear infection
*Klebsiella oxytoca*	A22	Cat	Skin infection
	I3	Cat	Corpse
	D22-B	Cat	Skin infection
	H9-B	Dog	Skin infection
	I1	Dog	Corpse

**Table 2:** Antimicrobial activity of G23.EE, expressed by MIC values (mg/mL), against a group of bacterial strains isolated from companion animals.

Bacterial Species	Isolate	Origin of the Isolate	MIC (mg/mL)
Gram-positive			
*Enterococcus faecalis*	406	Spleen (corpse)	>8.0
	463	Liver (corpse)	8.0
*Enterococcus faecium*	414	Spleen (corpse)	1.5
	434	Blood culture (corpse)	1.5
*Streptococcus canis*	786	Blood culture (corpse)	>8.0
*Staphylococcus arlettae*	CRIO-2	Ear infection	1.5
*Staphylococcus aureus*	J63 *	Skin infection	1.0
*Staphylococcus condimenti*	V16-A	Ear infection	1.0
*Staphylococcus felis*	J35-B	Skin infection	0.5
*Staphylococcus hominis*	A11 *	Skin infection	0.5
*Staphylococcus pseudintermedius*	A17-2 *	Skin infection	0.5
	A45	Ear infection	0.5
	J6-B	Ear infection	1.5
	J43-B *	Skin infection	1.5
*Staphylococcus schleiferi*	V3-B	Skin infection	1.5
*Staphylococcus sciuri*	J14-B	Skin infection	1.5
*Staphylococcus simulans*	V9-A	Skin infection	0.5
Gram-negative			
*Escherichia coli*	A36	Gastrointestinal	>8.0
	A28	Skin infection	
	D22-A	Skin infection	
	J6-A	Ear infection	
*Klebsiella oxytoca*	A22	Skin infection	>8.0
	I3	Corpse	
	D22-B	Skin infection	
	H9-B	Skin infection	
	I1	Corpse	

* Note: MDR strains.

## Data Availability

The original contributions presented in this study are included in the article/Supplementary Materials. Further inquiries can be directed toward the corresponding author(s).

## Supplementary Materials

The following supporting information can be downloaded at: https://www.mdpi.com/article/10.3390/pathogens14080826/s1, Table S1: Antimicrobial activity of the G23.EE, expressed as MIC values (mg/mL), against the set of bacteria used as susceptibility indicator strains for assessment of G23.EEs antimicrobial properties.
