# Supplementary material for: In Vitro Investigation of the Antimicrobial Properties of Gerês Propolis in Bacteria Isolated from Companion Animals and Safety Profile Characterization Using the Galleria mellonella Model
Source: Pathogens. 2025 Aug 21;14(8):826. doi: 10.3390/pathogens14080826 (PMC12389429; doi:10.3390/pathogens14080826)
Supplement: Supplementary file 1 [file pathogens-14-00826-s001.zip › pathogens-3759893-supplementary.pdf]

pathogens

MDPI

Type of the Paper (Article)

In vitro investigation of the antimicrobial properties of Gerês propolis in bacteria isolated from companion animals and safety profile characterization using the *Galleria mellonella* model

Rafael Rodrigues [1,†], Rui Almeida [1,†], Soraia C. V. Rodrigues [1], Joana Castro [2,3], Ricardo Oliveira [2,4,5], Nuno Mendes [1], Carina Almeida [2,3,4,5], Sónia Silva [2,3,6], Daniela Araújo [2,3,*] and Cristina Almeida-Aguiar [1,7,*]

Supplementary Materials

Table S1. Antimicrobial activity of the G23.EE, expressed as MIC values (mg/mL), against the set of bacteria used as susceptibility indicator strains for assessment of G23.EEs antimicrobial properties.

**Strain**	**MIC**
**Gram-positive bacteria**	**(mg/mL)**
Bacillus subtillis	0.1
Bacillus cereus	0.75
Bacillus megaterium	1.0
Staphylococcus aureus methicillin sensitive (MSSA)	2.0
Staphylococcus aureus methicillin resistant (MRSA)	> 2.0
Staphylococcus epidermidis	1.0
**Gram-negative bacteria**	
Escherichia coli	>2.0
